# Molecular characterisation of rotavirus strains detected during a clinical trial of the human neonatal rotavirus vaccine (RV3-BB) in Indonesia

**DOI:** 10.1016/j.vaccine.2018.08.027

**Published:** 2018-09-18

**Authors:** Daniel Cowley, Hera Nirwati, Celeste M. Donato, Nada Bogdanovic-Sakran, Karen Boniface, Carl D. Kirkwood, Julie E. Bines

**Affiliations:** a Enteric Virus Group, Murdoch Children’s Research Institute, Parkville, Victoria, Australia; b Rotavirus Program, Murdoch Children’s Research Institute, Parkville, Victoria, Australia; c Department of Paediatrics, The University of Melbourne, Parkville, VIC, Australia; d Department of Microbiology, Faculty of Medicine, Universitas Gadjah Mada, Yogyakarta, Indonesia; e Biomedicine Discovery Institute and Department of Microbiology, Monash University, Melbourne, Victoria, Australia; f Department of Gastroenterology and Clinical Nutrition, Royal Children’s Hospital, Parkville, Victoria, Australia

**Keywords:** Rotavirus, Diarrhoea, Neonatal, Vaccine

## Abstract

**Background:**

The RV3-BB human neonatal rotavirus vaccine aims to provide protection from severe rotavirus disease from birth. The aim of the current study was to characterise the rotavirus strains causing gastroenteritis during the Indonesian Phase IIb efficacy trial.

**Methods:**

A randomized, double-blind placebo-controlled trial involving 1649 participants was conducted from January 2013 to July 2016 in Central Java and Yogyakarta, Indonesia. Participants received three doses of oral RV3-BB vaccine with the first dose given at 0–5 days after birth (neonatal schedule), or the first dose given at ∼8 weeks after birth (infant schedule), or placebo (placebo schedule). Stool samples from episodes of gastroenteritis were tested for rotavirus using EIA testing, positive samples were genotyped by RT-PCR. Full genome sequencing was performed on two representative rotavirus strains.

**Results:**

There were 1110 episodes of acute gastroenteritis of any severity, 105 episodes were confirmed as rotavirus gastroenteritis by EIA testing. The most common genotype identified was G3P[8] (90/105), the majority (52/56) of severe (Vesikari score ≥11) rotavirus gastroenteritis episodes were due to the G3P[8] strain. Full genome analysis of two representative G3P[8] samples demonstrated the strain was an inter-genogroup reassortant, containing an equine-like G3 VP7, P[8] VP4 and a genogroup 2 backbone I2-R2-C2-M2-A2-N2-T2-E2-H2. The complete genome of the Indonesian equine-like G3P[8] strain demonstrated highest genetic identity to G3P[8] strains circulating in Hungary and Spain.

**Conclusions:**

The dominant circulating strain during the Indonesian Phase IIb efficacy trial of the RV3-BB vaccine was an equine-like G3P[8] strain. The equine-like G3P[8] strain is an emerging cause of severe gastroenteritis in Indonesia and in other regions.

## Highlights

• Equine-like G3P[8] the major cause of gastroenteritis during RV3-BB efficacy trial.
• The Indonesian equine-like G3P[8] strain was genetically similar to Hungarian and Spanish strains.
• Equine-like G3P[8] strain is an emerging cause of gastroenteritis in Indonesia.

## 1 Introduction

Rotavirus is the most common cause of severe gastroenteritis in children under five years of age [1]. The currently available rotavirus vaccines have been introduced into the national immunisation programs of 92 countries globally and reduced hospital admissions and child mortality from gastroenteritis [2], [3], [4], [5]. Despite this success, several barriers to global vaccine implementation exist, including cost and sub-optimal efficacy in low-income countries [6]. The human neonatal rotavirus vaccine, RV3-BB, is in clinical development with a birth dose vaccination schedule and is proposed to address some of these barriers. The RV3-BB vaccine is based on a naturally attenuated asymptomatic neonatal G3P[6] rotavirus strain, first identified in Melbourne obstetric hospitals in the 1970s [7]. A randomized, placebo-controlled trial to evaluate the efficacy of an oral human-strain neonatal rotavirus vaccine (RV3-BB) was recently completed in central Java and Yogyakarta, Indonesia [8]. Vaccine efficacy against severe rotavirus gastroenteritis from 2 weeks after dose 3 and to 18 months of age was 63% in the combined vaccine group (95% confidence interval [CI] 34, 80; p < 0.001), 75% in the neonatal vaccine group (95% CI 44, 91; p < 0.001) and 51% in the infant vaccine group (95% CI 7, 76; p = 0.03). The vaccine was also found to be immunogenic and well-tolerated when administered in either the neonatal or infant schedules.

The rotavirus strains which circulate in the human population demonstrate significant genetic diversity. The rotavirus genome is comprised of 11 segments of double stranded RNA, encoding six structural (VP1-4, VP6, VP7) and six non-structural proteins (NSP1-5/6) [9]. The segmented genome facilitates reassortment between strains, allowing both intra- and inter-genogroup reassortment. Continued genetic variation by sequential point mutations and zoonotic transmission of novel animal strains also increases the genetic diversity within circulating rotavirus strains causing human infection. A genotype classification system based on capsid genes VP7 and VP4 is used in molecular epidemiology of rotavirus strains denoting the G-type (glycoprotein) and P-type (protease sensitive) respectively [9]. Whole genome classification is also used, the nomenclature Gx-P[x]-Ix-Rx-Cx-Mx-Ax-Nx-Tx-Ex-Hx represents the genotypes of VP7-VP4-VP6-VP1-VP2-VP3-NSP1-NSP2-NSP3-NSP4-NSP5/6 respectively [10]. Currently there are 35 G, 50 P, 26 I, 21 R, 19 C, 19 M, 30 A, 21 N, 21 T, 27 E and 21 H types [11]. There are two major genotype constellations of human rotaviruses, termed genogroup 1 (G1-P[8]-I1-R1-C1-M1-A1-N1-T1-E1-H1), genogroup 2 (G2-P[4]-I2-R2-C2-M2-A2-N2-T2-E2-H2) [12].

A more dynamic and diverse rotavirus strain population has been observed in the vaccine era [13], [14]. For rotavirus vaccines to be effective they must provide protection against multiple circulating genotypes. Strain diversity may also be a factor in vaccine effectiveness in low- and middle-income countries, which can have a higher diversity of strains and distinct dominant genotypes compared to high-income settings [15]. Therefore, understanding the genetic diversity of the rotavirus strains causing gastroenteritis during the RV3-BB vaccine trial will provide valuable insights for future implementation of this vaccine. During the phase IIb efficacy trial, study participants were followed for episodes of gastroenteritis from birth to 18 months of age. In the present study, we sought to characterise the genetic diversity of strains causing acute gastroenteritis during the trial of the RV3-BB in Indonesia.

## 2 Method and materials

### 2.1 Study design and participants

The study design and recruitment for the Phase IIb efficacy, safety and immunogenicity trial of the RV3-BB vaccine has been previously described [8]. Briefly, a randomized, double-blind placebo-controlled trial involving 1649 participants was conducted from January 2013 to July 2016 in primary health centres and hospitals in Klaten, Central Java, and Sleman, Yogyakarta, Indonesia. Eligible infants (healthy, full term babies 0–5 days of age, birth weight of 2.5–4.0 kg) were randomized into one of three groups (neonatal vaccine group, infant vaccine group, or placebo group) in a 1:1:1 ratio according to a computer-generated code (block size = 6) which was stratified by province. The trial protocol was approved by the ethics committees of Universitas Gadjah Mada, Royal Children's Hospital Melbourne and National Agency of Drug and Food Control, Republic of Indonesia.

During the recruitment process, 2405 pregnant women gave antenatal preliminary consent, 1649 were randomized, 549 to neonatal vaccine schedule, 550 to infant vaccine schedule, 550 to placebo schedule. The analysis of vaccine efficacy was performed on per protocol (n = 1513) and intention to treat (ITT) (n = 1649) populations followed for severe episodes of rotavirus gastroenteritis occurring from two weeks post investigational product (IP) dose 4 to 18 months of age [8]. To characterise all rotavirus positive cases the current genotype analysis was performed on the ITT population, and included episodes of rotavirus gastroenteritis of any severity that occurred from administration of the birth dose until 18 months of age.

The investigational product (IP) consisted of RV3-BB vaccine (8.3–8.7 × 10^6^ FCFU/ml) or Placebo (cell culture medium, DMEM). RV3-BB clinical trial lots were prepared at Meridian Life Sciences (Memphis, USA) to a titre of 8.3–8.7 × 10^6^ FFU/mL in serum free media supplemented with 10% sucrose. Placebo contained the same media with 10% sucrose and was visually indistinguishable. Vials were stored at −70 °C until thawed within 6 h prior to administration.

### 2.2 Sample collection and processing

The participant’s parent(s)/guardian(s) were asked to collect at least two faecal samples per diarrhoea episode, from separate stools. Samples were obtained using faecal spatulas to scrape at least two scoops of faeces from infants’ skin or nappy, which was then stored in a faecal specimen container. If faeces were too liquid and a specimen was unable to be obtained, plastic film inside the nappy was used to assist sample collection or the whole nappy was collected for analysis. Stool samples were stored at 2–10 °C within 4 h of collection, and transported to the Universitas Gadjah Mada microbiology laboratory within 24 h. Upon receipt at laboratory, stool samples were aliquoted and stored at −70 °C.

### 2.3 Rotavirus antigen testing

Stool samples were tested for rotavirus antigen using the commercial rotavirus enzyme immunoassay (EIA) ProSpecT (Oxoid, Ltd, UK), as per manufacturer’s instructions. Severe rotavirus gastroenteritis was defined as rotavirus gastroenteritis with a modified Vesikari score of ≥11 [8].

### 2.4 Rotavirus genotyping

Viral RNA was extracted from 10% to 20% w/v faecal extracts of each specimen using the Viral Nucleic Acid Extraction Kit II (Geneaid) according to the manufacturer’s instructions. The rotavirus G and P genotype were determined for each sample by the application of independent hemi-nested multiplex reverse transcription polymerase chain reaction (RT-PCR) assays. The first-round RT-PCR assays were performed using the Superscript III One-Step RT-PCR (Invitrogen), using VP7 conserved primers 9Con1-L and VP7R, or VP4 conserved primers Con-2 and Con-3 [16], [17]. The second-round genotyping PCR reactions were conducted using specific oligonucleotide primers for G types 1, 2, 3, 4 and 9 or P types [4], [6], [8], [9], [10] [18]. The G and P genotype of each sample was assigned using agarose gel analysis of second-round PCR products.

### 2.5 Polyacrylamide gel electrophoresis

G3P[8] samples with adequate volume were selected for analysis. The 11 segments of rotavirus dsRNA were separated on 10% (w/v) polyacrylamide gel with 3% (w/v) polyacrylamide stacking gel at 25 mA for 16 h. The genome migration patterns (electropherotypes) were visualized by silver staining according to the established protocol [19], [20].

### 2.6 Amplification of complete rotavirus genomes

The 11 gene segments were reverse transcribed and amplified by PCR using the PrimerScript High Fidelity RT-PCR Kit (Takara, Japan) as previously described [21]. Primers used in the amplification of the 11 gene segments are detailed elsewhere [22].

### 2.7 Nucleotide sequencing

PCR amplicons were purified using the Wizard SV Gel for PCR Clean-Up System (Promega, USA) according to the manufacturer’s protocol. Purified cDNA was sequenced using an ABI PRISM BigDye Terminator Cycle Sequencing Reaction Kit (Applied Biosystems, Foster City, CA, USA) in an Applied Biosystems 3730xl DNA Analyzer (Applied Biosystems, Foster City, CA, USA). Primer walking was employed to cover the complete nucleotide sequence of each gene [22].

### 2.8 Phylogenetic analysis

Contiguous DNA sequence files were constructed utilizing Sequencher software (version 5.0.1; Gene Codes). The genotypes of each of the 11 genome segments were determined using the online RotaC v2.0 rotavirus genotyping tool (http://rotac.regatools.be) in accordance with the recommendations of the Rotavirus Classification Working Group (RCWG) [23]. Nucleotide similarity searches were performed using the BLAST server on the GenBank database. The nucleotide and deduced amino acid sequences of each gene were compared with sequences available in GenBank possessing the entire open reading frame using the Virus Variation resource [24]. Multiple nucleotide and amino acid alignments were constructed using the MUSCLE algorithm in MEGA 6.0 [25]. Nucleotide and amino acid distance matrixes were calculated using the p-distance algorithm in MEGA 6.0. The optimal evolutionary model was selected based upon the Akaike information criterion (corrected) (AICc) ranking implemented in jModelTest [26]. Maximum-likelihood phylogenetic trees using the selected models of nucleotide substitution HKY + G_G4_ (VP7) and GTR + G_G4_ (VP4) were reconstructed using MEGA 6.0 [25], [27]. The robustness of branches was assessed by bootstrap analysis using 1000 pseudo-replicate runs. The mVISTA software was used to visualize the comparative sequence similarities of concatenated whole genome of genetically related strains [28].

### 2.9 Accession numbers

Nucleotide sequences for RVA/Human-wt/IDN/D006389b/2014/G3P[8] and RVA/Human-wt/IDN/D009617g/2015/G3P[8] were deposited in GenBank under the accession numbers MH704718-MH704739.

## 3 Results

### 3.1 Number of gastroenteritis episodes and stool samples

During the study 1649 participants were randomized and included in the ITT population. Of the 1649 participants, 1640 received at least one dose of IP and 1588 were followed to 18 months of age. From the birth dose until 18 months of age, 701/1649 (42.5%) participants had at least one episode of gastroenteritis of any severity. There were 1110 unique episodes of gastroenteritis, multiple episodes were recorded in a subset of participants. Rotavirus enzyme immunoassay (EIA) antigen testing was performed on 1246 stool samples. Testing was performed on the first sample collected per diarrhoea episode, however in a limited number of episodes multiple samples were tested. There were 105/1110 (9.5%) episodes of gastroenteritis that were rotavirus positive. There were 23/105 (21.9%) episodes in the neonatal vaccine schedule, 29/105 (27.6%) in the infant vaccine schedule and 53/105 (50.4%) in the placebo schedule.

### 3.2 Genotyping of rotavirus positive gastroenteritis episodes

Rotavirus genotyping was performed on the 105 rotavirus positive gastroenteritis episodes. The most common genotype identified was G3P[8], this genotype represented 85.7% (90/105) of rotavirus strains (Table 1). Genotype G3P[8] rotavirus strains were identified in participants from each vaccination schedule, the majority (52/56) of severe rotavirus gastroenteritis cases (Vesikari score ≥ 11) were due to a G3P[8] strain. The other genotypes identified included G2P[6] (5/105, 4.7%), G1P[8] (1/105, <1%) and G3P[6] (1/105, <1%). None of the G2P[6] or G1P[8] strains were associated with severe rotavirus gastroenteritis. A small number of samples could only be partially genotyped (7/105, 6.6%) or had a mixed genotype (1/105, <1%).

**Table 1:** Rotavirus genotypes identified in stool sample collected for cases of acute gastroenteritis.

Genotype	No. (%)	No. per schedule			Vesikari score	
		Neonatal	Infant	Placebo	Not severe (<11)	Severe (≥11)
G3P[8]	90 (85.7%)	20	23	47	38	52
G2P[6]	5 (4.7%)	0	3	2	5	0
G3P[6]	1 (<1%)	0	0	1	0	1
G1P[8]	1 (<1%)	1	0	0	1	0
Mixed (G2,3P[6])	1 (<1%)	1	0	0	1	0
Partial Type (GXP[8]; G3P[X])	7 (6.6%)	1	3	3	4	3
Total	105	23	29	53	49	56

Neonate and Infant schedule participants received RV3-BB vaccine according the dosing schedule described in Section 2. Severe gastroenteritis defined by a Vesikari score ≥11.

### 3.3 Whole genome analysis of representative G3P[8] strains

Polyacrylamide gel electrophoresis was performed on a subset G3P[8] strains with sufficient sample volume (38/90). These strains were collected from the Klaten district of central Java and Sleman district of Yogyakarta throughout conduct of the trial. Strains with a visible electropherotype (27/90) had similar profiles, however there were several circulating variants with differences in the migration of the NSP2, NSP4 and NSP5/6 RNA segments (data not shown). Sequencing of the VP7 gene (nt 72–914) from 44/90 G3P[8] strains demonstrated 98.7–100% nucleotide and 97.5–100% amino acid identity. BLAST search and phylogenetic analysis of these VP7 genes demonstrated that they clustered with previously described human equine-like G3P[8] strains (data not shown) [22].

Whole genome analysis was performed on two G3P[8] strains, one from the Klaten district collected in 2014, RVA/Human-wt/IDN/D006389b/2014/G3P[8] and one from the Sleman district collected in 2015, RVA/Human-wt/IDN/D009617g/2015/G3P[8]. These two strains demonstrated high nucleotide identity for all gene segments (99.1–99.9%), with the genome constellation G3-P[8]-I2-R2-C2-M2-A2-N2-T2-E2-H2 identified.

**Fig. 1:**
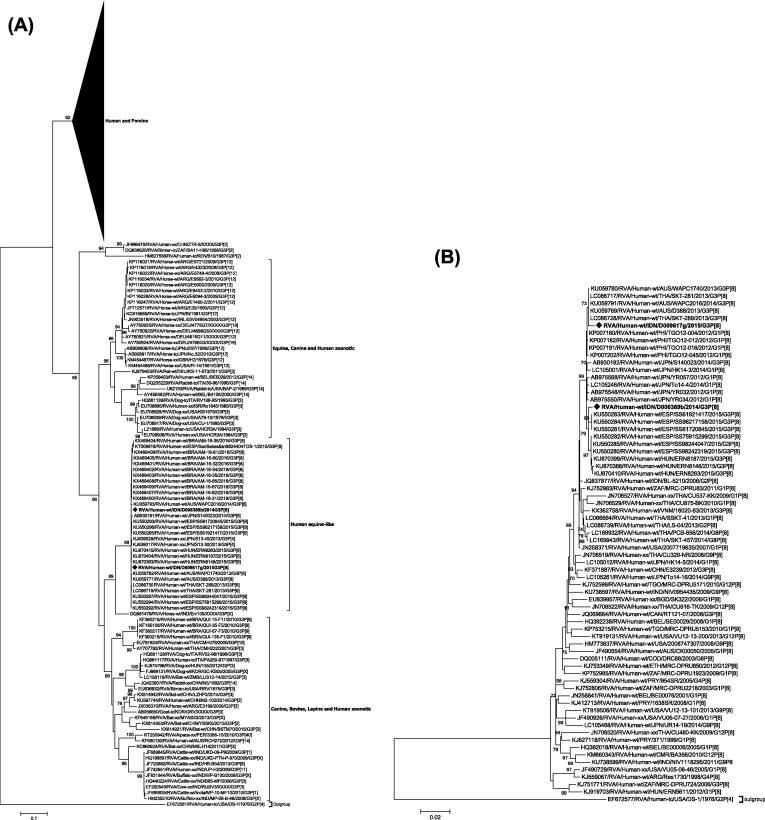
Phylogenetic tress constructed from the nucleotide sequences of (A) VP7 and (B) VP4 genes of rotavirus strains D006389b and D009617g with other group A rotavirus strains representing the G3 and P[8] genotypes respectively. The position of strains D006389b and D009617g are indicated by a ♦ symbol and all strains from this study are in bold. Bootstrap values ≥ 70% are shown. Scale bar shows substitutions per site. The nomenclature of all the rotavirus strains indicates the rotavirus group, species isolated from, country of strain isolation, the common name, year of isolation, and the genotypes for genome segment 9 and 4 as proposed by the RCWG [49].

The VP7 genes of RVA/Human-wt/IDN/D006389b/2014/G3P[8] and RVA/Human-wt/IDN/D009617g/2015/G3P[8] clustered in a lineage divergent to the majority of human and porcine G3 strains. This lineage comprised of strains derived from numerous animal species (Fig. 1A). Furthermore, the VP7 genes were distinct from the G3 sequence of the RV3-BB vaccine which clusters in the main human/porcine lineage, sharing only 82.4% nucleotide and 92.1% amino acid identity. Both Indonesian strains clustered with contemporary human equine-like G3P[8] strains from Australia, Brazil, Japan, Spain, Thailand sharing > 99.1% nucleotide identity and the equine strain RVA/Horse-wt/IND/Erv105/2004-05/G3P[X] with 90.6% nucleotide identity. The Indonesian strains clustered within a lineage that contained two additional discrete sub-lineages that were supported by strong bootstrap values, one comprised of equine strains, and one primarily comprised of canine, bovine, and lapine strains and human strains predominantly derived from zoonotic transmission.

The VP4 genes of RVA/Human-wt/IDN/D009617g/2015/G3P[8] clustered with the Australian strains RVA/Human-wt/AUS/WAPC2016/2014/G3P[8], RVA/Human-wt/AUS/D388/2013/G3P[8] and RVA/Human-wt/AUS/WAPC1740/2013/G3P[8] and the Thai strains RVA/Human-wt/THA/SKT-281/2013/G3P[8] and RVA/Human-wt/THA/SKT-289/2013/G3P[8] (Fig. 1B). The VP4 gene of RVA/Human-wt/IDN/D006389b/2014/G3P[8] clustered with multiple Spanish strains including RVA/Human-wt/ESP/SS61720845/2015/G3P[8] and Hungarian strains including RVA/Human-wt/HUN/ERN8263/2015/G3P[8]. Both VP4 genes clustered within a sub-lineage predominantly comprised of strains from Spain, Hungary, the Philippines, Vietnam and Japan sharing > 99.3% nucleotide identity.

**Fig. 2:**
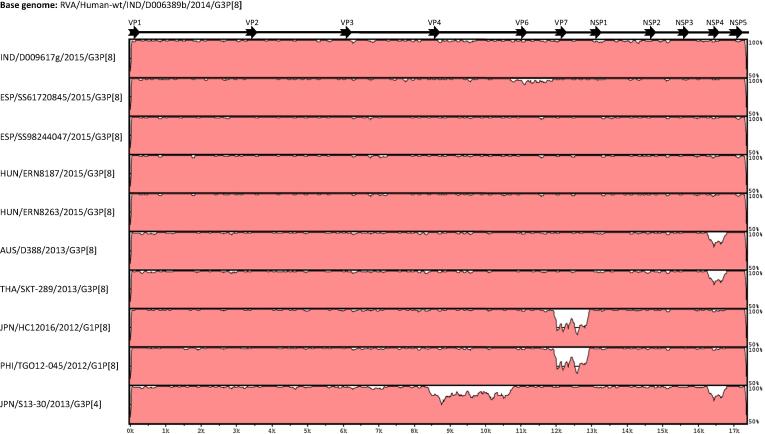
The nucleotide sequence similarities of concatenated genome of D006389b were compared to strains D009617g, SS61720845, SS98244047, ERN8187, ERN8263, D388, SKT-289, HC12016, TGO12-045, S13-30. The date and location of identification of each of the comparator strains is included on the right axis. Rotavirus genome segment is shown in the top scale.

The concatenated genomes of RVA/Human-wt/IDN/D006389b/2014/G3P[8] and RVA/Human-wt/IDN/D009617g/2015/G3P[8] were compared to equine-like G3 reassortant strains identified in Hungary, Spain, and Thailand and to the G1P[8] inter-genogroup reassortant strains identified in Japan and the Philippines (Fig. 2). Across all gene segments, RVA/Human-wt/IDN/D006389b/2014/G3P[8] and RVA/Human-wt/IDN/D009617g/2015/G3P[8] exhibited the highest overall genetic identity to European strains, including the Spanish strains RVA/Human-wt/ESP/SS98244047/2015/G3P[8], RVA/Human-wt/ESP/SS61720845/2015/G3P[8] and the Hungarian strains RVA/Human-wt/HUN/ERN8187/2015/G3P[8] and RVA/Human-wt/HUN/ERN8263/2015/G3P[8]. With the exception of the NSP4 genes, the Australian and Thai strains RVA/Human-wt/AUS/D388/2013/G3P[8] and RVA/Human-wt/THA/SKT-289/2013/G3P[8] shared a highly conserved genome with RVA/Human-wt/IDN/D006389b/2014/G3P[8] and RVA/Human-wt/IDN/D009617g/2015/G3P[8]. Similarly, with the exception of the VP7 gene, RVA/Human-wt/IDN/D006389b/2014/G3P[8] and RVA/Human-wt/IDN/D009617g/2015/G3P[8] shared conserved genome segments with Japanese RVA/Human-wt/JPN/HC12016/2012/G1P[8] and RVA/Human-wt/PHI/TGO12-045/2012/G1P[8] from the Philippines.

## 4 Discussion

The human neonatal vaccine RV3-BB provided protection against severe gastroenteritis in a Phase IIb efficacy trial conducted in Yogyakarta and Central Java, Indonesia [8]. Here, we report that the majority rotavirus gastroenteritis cases identified during the Phase IIb trial were caused by an equine-like G3P[8] strain.

Full genome analysis on the Indonesia G3P[8] strain demonstrated it was an inter-genogroup reassortant, containing an equine-like G3 VP7, a P[8] VP4 gene and a genogroup 2 backbone I2-R2-C2-M2-A2-N2-T2-E2-H2. This strain has not been previously reported in strain surveillance conducted in Indonesia [29], [30]. The genomes of the Indonesian equine-like G3P[8] strains were most similar to strains detected in Spain and Hungry in 2015 [31], [32]. These inter-genogroup reassortant strains share a similar genogroup 2 backbone with G1P[8] and G3P[4] strains first associated with multiple outbreaks in 2012–2013 in Japan [33], [34]. The Indonesia equine-like G3P[8] strains also demonstrated high genetic similarity to an equine-like G3P[8] inter-genogroup reassortant strain that emerged in Australia and was the dominant strain in Australian children with severe rotavirus gastroenteritis in 2013 [22]. Equine-like G3P[8] strains with the same genome constellation have also recently been reported in other countries in Asia and South America [35], [36]. A recent report from Surabaya, Indonesia (published while our work was under review), identified two distinct equine-like G3P[8] strains circulating in 2015–2016 [37]. This data correlates with the multiple equine-like G3P[8] strains we identified by PAGE and VP7 sequence analysis, suggesting diversity in the circulating equine-like G3P[8] strains in Indonesia. Whilst we are not able to describe the precise origins of these strains, the detection of equine-like inter-genogroup G3P[8] strains in Indonesia adds further evidence to the global importance of this strain as a cause of gastroenteritis.

The mechanisms of protection following vaccination with RV3-BB and other rotavirus vaccines remains unclear. The rotavirus strains which circulate demonstrate considerable genetic variation from year to year, as well as within and between countries [10], [12], [13], [38]. Therefore, to be effective rotavirus vaccines must provide heterotypic protection against a diverse population of strains. Following infection antibody responses to the capsid proteins VP7, VP4, VP6 and VP2 [39], [40], [41], [42], and non-structural proteins NSP2 and NPS4 [39], [41], [43], [44] have been reported. Broadly heterotypic antibodies are directed at VP4 (VP5* and VP8*), VP7 and VP6 proteins [45] indicating that these proteins contain cross reactive epitopes. In addition, conserved CTL epitopes have also been described in the VP3 protein [46]. It is probable that one or more of these cross-reactive epitopes contribute to heterotypic protection. Due to the predominance of the equine-like G3P[8] in our study we are unable to assess the heterotypic protection provided by RV3-BB. However, the equine-like G3P[8] is genetically distinct when compared to RV3-BB and the previously circulating human G3 strains [22]. The VP7 genes of the Indonesian equine-like G3P[8] and RV3-BB share only 82.4% nucleotide and 92.1% amino acid identity. Furthermore, RV3-BB has typical genogroup 1 genome constellation G3-P[6]-I1-R1-C1-M1-A1-N1-T1-E1-H1 [47], which is distinct to the equine-like G3-P[8]-I2-R2-C2-M2-A2-N2-T2-E2-H2 constellation which we report here. This data suggests that the protection provided by RV3-BB in the Indonesian trial was cross protective and likely not solely dependent on homotypic responses. The strong heterotypic serological responses to community strains (G1, G2) provided by the parental RV3 strain further supports this hypothesis [7], [48]. However, additional studies are required to demonstrate the degree of heterotypic protection provided by RV3-BB.

To conclude, we characterized a novel equine-like G3P[8] strain circulating in Indonesia during the conduct of the Phase IIb RV3-BB efficacy trial. This strain was genetically similar to the equine-like G3P[8] inter-genogroup reassortant strain which has emerged globally since 2013.

## 5 Contributors

DC, HN, CDK and JEB were involved in the conception and design of the study. HN, DC, KB, NB were involved in the acquisition of data. DC, CD and JEB were involved in the analysis and interpretation of data. DC wrote the manuscript. All authors approved the final version of the manuscript.
